# Long-Term Humoral Immune Response in Persons with Asymptomatic or Mild SARS-CoV-2 Infection, Vietnam

**DOI:** 10.3201/eid2702.204226

**Published:** 2021-02

**Authors:** Huynh Kim Mai, Nguyen Bao Trieu, Trinh Hoang Long, Hoang Tien Thanh, Nguyen Dinh Luong, Le Xuan Huy, Lam Anh Nguyet, Dinh Nguyen Huy Man, Danielle E. Anderson, Tran Tan Thanh, Nguyen Van Vinh Chau, Guy Thwaites, Lin-Fa Wang, Le Van Tan, Do Thai Hung

**Affiliations:** Pasteur Institute, Nha Trang City, Vietnam (H.K. Mai, N.B. Trieu, T.H. Long, H.T. Thanh, N.D. Luong, L.X. Huy, D.T. Hung);; Oxford University Clinical Research Unit, Ho Chi Minh City, Vietnam (L.A. Nguyet, T.T. Thanh, G. Thwaites, L.V. Tan);; Hospital for Tropical Diseases, Ho Chi Minh City (D.N.H. Man, N.V.V. Chau); Duke-NUS Medical School, Singapore (D.E. Anderson, L.-F. Wang);; Centre for Tropical Medicine and Global Health, Nuffield Department of Medicine, University of Oxford, Oxford, UK (G. Thwaites);; SingHealth Duke-NUS Global Health Institute, Singapore (L.-F. Wang)

**Keywords:** asymptomatic coronavirus disease, COVID-19, immune response, neutralizing antibodies, respiratory infections, SARS-CoV-2, SARS-CoV-2 spike protein, viruses

## Abstract

Antibody response against nucleocapsid and spike proteins of SARS-CoV-2 in 11 persons with mild or asymptomatic infection rapidly increased after infection. At weeks 18–30 after diagnosis, all remained seropositive but spike protein–targeting antibody titers declined. These data may be useful for vaccine development.

Severe acute respiratory syndrome coronavirus 2 (SARS-CoV-2) is the causative agent of the coronavirus disease (COVID-19) pandemic (*1*). Effective vaccines are vital for mitigating the impact of the pandemic. As such, synthesizing a long-term humoral immune response to SARS-CoV-2 remains essential to developing and implementing a SARS-CoV-2 vaccine. We report a longitudinal study of 11 persons with SARS-CoV-2 infection in Vietnam, in which we monitored antibody responses for up to 30 weeks after infection. 

We included patients with a confirmed SARS-CoV-2 infection admitted to a COVID-19 treatment center in central Vietnam during January–March 2020. To enable long-term follow-up, we excluded all short-term visitors. We collected information from each participant about clinical status, travel history, contacts with persons with confirmed cases, and personal demographics. For plasma collection, we applied a flexible sampling schedule encompassing 30 weeks after diagnosis, stratified by collection at 1, 2–3, 4–7, and ≥18 weeks after diagnosis.

We measured antibodies against 2 main immunogens of SARS-CoV-2, the nucleocapsid (N) and spike (S) proteins, by using 2 well-validated sensitive and specific serologic assays, Elecsys Anti–SARS-CoV-2 assay (Roche, https://diagnostics.roche.com) (*2*) and SARS-CoV-2 Surrogate Virus Neutralization Test (sVNT) (GenScript, https://www.genscript.com) (*3*). The former is an electrochemiluminescence immunoassay that uses recombinant N protein for qualitative detection of pan Ig, including IgG, against SARS-CoV-2. The latter is a surrogate assay for measuring receptor-binding domain–targeting neutralizing antibodies (RBD-targeting NAbs) (*3*,*4*), in principle a blocking ELISA that quantifies antibodies that block the receptor–RBD interaction (*3*). Our study forms part of the national COVID-19 response and was approved by the institutional review board of the Pasteur Institute in Nha Trang, Vietnam.

During the study period, there were a total of 23 patients with confirmed SARS-CoV-2 infection in central Vietnam. Ten were tourists and were thus excluded from the study. Of the remaining 13, a total of 11 consented to participate in this study. Among study participants, 6 were female and 5 were male; the age range was 12–64 years (Table). Seven experienced mildly symptomatic infection and did not require supplemental oxygen during hospitalization; 4 were asymptomatic. Before becoming ill, 3 had traveled to a SARS-CoV-2–endemic country, including patients 2 and 3, who had traveled to Malaysia and patient 4 had traveled to the United States. Patient 4 transmitted the virus to 6 of her contacts, including 4 family members and 2 employees. Of these, 2 transmitted the virus to another family member (Table; Appendix Figure). 

**Table:** Demographics, travel history, contact history, clinical status, and outcome for participants in study of long-term humoral immune response in persons with asymptomatic or mild SARS-CoV-2 infection, Vietnam, 2020*

Patient no.†	Age, y/sex	Province	Presumed exposure	Symptoms developed	Diagnosed	Presumed incubation period, d	Recent travel history	Contact with confirmed patient	Clinical status	Hospital stay, d
1	25/F	Khanh Hoa	Jan 14	Jan 18	Jan 24	4	None	1 of first 2 cases in Vietnam	Sympt	11
2	42/M	Ninh Thuan	Feb 27–Mar 4	Mar 9	Mar 16	5–14	Malaysia	Unknown	Sympt	16
3	36/M	Ninh Thuan	Feb 27–Mar 4	Mar 13	Mar 17	9–15	Malaysia	Unknown	Sympt	15
4	51/F	Binh Thuan	Feb 22–29	Mar 5	Mar 9	7–14	USA	Unknown	Sympt	25
5‡	51/M	Binh Thuan	Mar 2–9	Mar 11	Mar 11	2–9	None	Husband of patient 4	Sympt	23
6‡	64/F	Binh Thuan	Mar 2–10	Asympt	Mar 10	5–8	None	Domestic worker of patient 4	Asympt	31
7‡	28/F	Binh Thuan	Mar 7	Asympt	Mar 10	3	None	Daughter-in-law of patient 4	Asympt	24
8‡	28/M	Binh Thuan	Mar 2–9	Mar 11	Mar 11	2–9	None	Son of patient 4	Sympt	23
9‡	47/F	Binh Thuan	Mar 3–8	Mar 11	Mar 11	3–8	None	Mother of patient 7	Sympt	23
10‡	37/F	Binh Thuan	Mar 3–8	Asympt	Mar 10	2–7	None	Staff of patient 4	Asympt	24
11‡	12/M	Binh Thuan	Mar 3–8	Asympt	Mar 11	2–7	None	Son of patient 10	Asympt	30

*All patients made a full recovery. No patients required oxygen. All patients were of Vietnamese nationality. First enrollment was on January 24, 2020, and last was on March 17, 2020. Last follow up was on August 13, 2020. Asympt, asymptomatic; sympt, symptomatic. †Patient numbers match those in Figure 1. ‡Patients from a cluster involving 3 household transmission chains (Appendix Figure).

We collected 43 plasma samples from 11 participants within 4 time ranges after diagnosis: <1 week (n = 10), weeks 2–3 (n=11), weeks 4–7 (n=11), and weeks 18–30 (n = 11). During the first week after diagnosis, 1 patient (1/10, 10%) had detectable RBD-targeting NAbs, and none had antibodies against N protein. In subsequent weeks, all (100%) participants tested positive by surrogate virus neutralization. Antibodies against N protein were detected in 10/11 (91%) of the samples collected between the second and third weeks after diagnosis and 11/11 (100%) samples collected at subsequent time points (Figure, panel A). 

**Figure:**
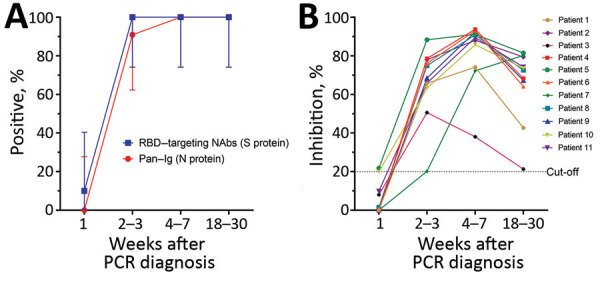
Antibody responses in 11 study participants, weeks 1–20 after PCR diagnosis of SARS-CoV-2 infection, Vietnam, 2020. A) Seroprevalence of SARS-CoV-2 among 11 COVID-19 patients. We followed testing protocols and the positive cutoff of 20% recommended in the Elecsys Anti–SARS-CoV-2 assay (Roche, https://diagnostics.roche.com) without any modification. Using these parameters, previous studies showed an excellent concordance between results from surrogate virus neutralization tests and conventional neutralizing antibody detection assays (*3*,*4*). Vertical bars denote 95% CIs. Graphs were created using GraphPad Prism version 8.0 (GraphPad software, https://www.graphpad.com). B) Kinetics of neutralizing antibodies measured by the surrogate neutralization assay (GenScript, https://www.genscript.com) with the 20% cutoff applied. We tested samples at 1:10 dilution as specified. Because of the limited availability of plasma samples, each sample was tested only once. RBD, receptor-binding domain; NAbs, neutralizing monoclonal antibodies; S, spike; N, nucleocapsid.

Previous studies have demonstrated that the inhibition percentage measured by surrogate virus neutralization tests correlates well with neutralizing antibody titers measured by conventional virus neutralization assays or plaque-reduction neutralization tests (*3*,*4*). In our study, the inhibition percentage was below the assay cutoff in all but 1 plasma sample taken during the first week after diagnosis and then rapidly increased above the assay cutoff at subsequent time points. At weeks 18–30 after diagnosis, the inhibition percentage declined but remained detectable (Figure, panel B).

We demonstrate that antibodies against 2 main structural proteins (S and N) of SARS-CoV-2 in patients with asymptomatic or mild infections were almost undetectable within the first week after diagnosis. Antibodies rapidly increased in subsequent weeks and peaked around weeks 4–7 before declining during the later phase of infection, consistent with previously reported findings (*2**,**5**–**7*). However, few studies have reported the persistence of long-term humoral immune response to SARS-CoV-2 up to 18–30 weeks after diagnosis (*5*), especially among mildly symptomatic or asymptomatic infected patients.

The titers of RBD-targeting NAbs, which are well correlated with those of neutralizing antibodies, decayed by weeks 18–30 after infection, suggesting that humoral immunity to SARS-CoV-2 infection may not be long lasting. Because neutralizing antibodies are recognized as a surrogate for protection (*7*–*9*), follow-up studies beyond this period are needed to more conclusively determine the durability of these long-term responses and their correlation with protection. 

Our collective findings offer insights into the long-term humoral immune response to SARS-CoV-2 infection. The data might have implications for COVID-19 vaccine development and implementation and other public health responses to the COVID-19 pandemic. 

Appendix Possible chain of transmission among contacts of early coronavirus disease patients in Vietnam, 2020.
