# Supplementary material for: Long-Term Humoral Immune Response in Persons with Asymptomatic or Mild SARS-CoV-2 Infection, Vietnam
Source: Emerg Infect Dis. 2021 Feb;27(2):663–6. doi: 10.3201/eid2702.204226 (PMC7853537; doi:10.3201/eid2702.204226)
Supplement: Appendix — Possible chain of transmission among contacts of early coronavirus disease patients in Vietnam, 2020. [file 20-4226-Techapp-s1.pdf]

Article DOI: https://doi.org/10.3201/eid2702.204226

# Long-Term Humoral Immune Response in Individuals with Asymptomatic or Mild SARS-CoV-2 Infection, Vietnam

## Appendix

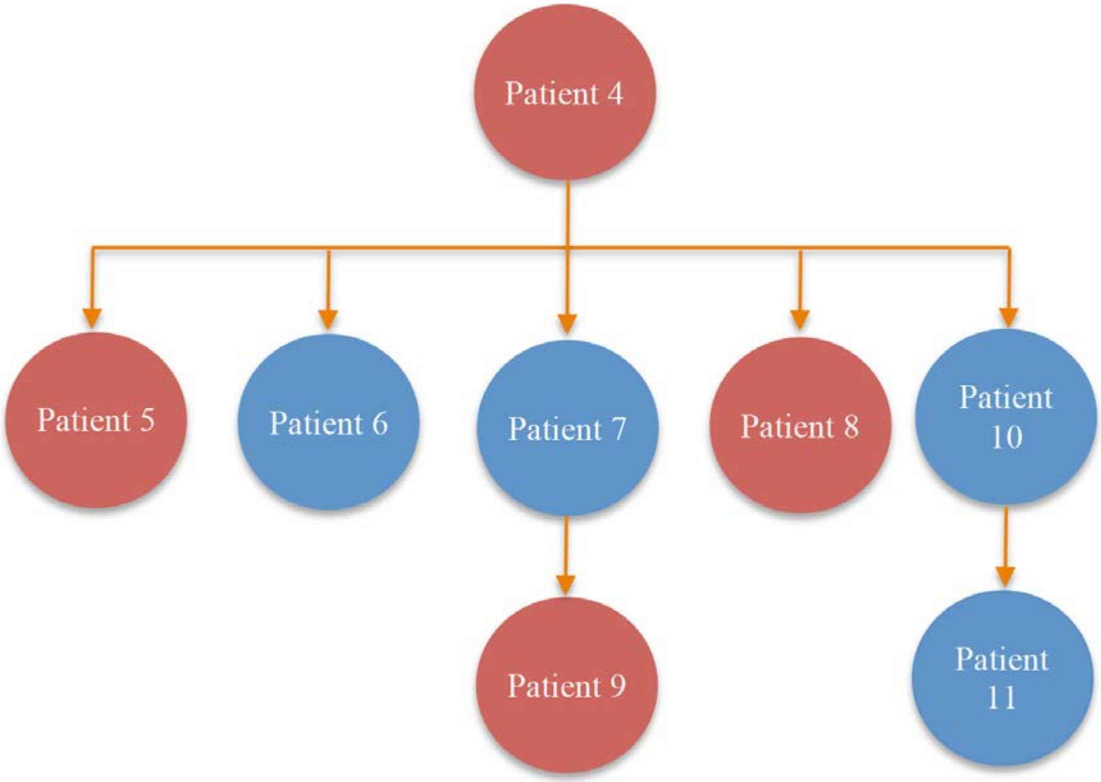


**Appendix Figure.** Illustration showing the possible transmission chains among persons from a cluster of cases involving members of 3 families. Red circles represent symptomatic patients. Blue circles represent asymptomatic patients. Patients are numbered using the same numbering system presented in the table and figure.
